# Multi-scale experimental and computational assessment of heat transfer behavior in compact short fin structures

**DOI:** 10.1038/s41598-026-43375-1

**Published:** 2026-03-11

**Authors:** Sampath Suranjan Salins, Aswathi Pallikkara Kuttiatoor, Gagan Pramod, Shiva Kumar

**Affiliations:** 1 Manipal Academy of Higher Education, Dubai Campus, PO 345050, Dubai, UAE; 2 Manipal Institute of Technology, Manipal Academy of Higher Education, Manipal, 576104 India

**Keywords:** Convective Heat Transfer, Thermal Analysis, temperature distribution, Fin Geometry, Experimentation, Energy science and technology, Engineering, Materials science, Physics

## Abstract

The present study presents a comparative analysis of temperature distribution and heat transfer performance of short fins using experimental, simulation and theoretical method. An experimental setup was developed to investigate square, circular, rectangular, trapezoidal, and triangular fins under a constant heat input of 30 W, with specimen lengths of 0–80 mm, temperatures of 77–50 °C, and natural convection conditions (ambient air at 25 °C, velocity 0.2 to 0.5 m/s). Results show that square fins achieved the highest heat transfer rate (5.32 W), efficiency (94.86%), and effectiveness (23.77), while cylindrical fins performed slightly lower. Among materials, mild steel with a rectangular cross-section demonstrated superior thermal performance, with a heat transfer rate of 5.42 W and fin effectiveness of 25.89. Thermal resistances for mild steel, stainless steel, cast iron, and titanium were 10.61, 32.55, 18.21, and 42.83 °C/W, respectively. The study reveals how short-fin geometry and material influence heat transfer, airflow, and turbulence under natural convection. The findings are scalable, providing guidance for designing heat sinks and cooling systems. By integrating experiments and simulations, the research identifies optimal fin configurations and uncovers localized thermal and flow behaviors, offering practical strategies for improved thermal management.

## Introduction

Fins are extended surfaces designed to improve heat transfer by increasing the surface area available for heat dissipation. These components are widely used across various industries, including transformers, automobiles, refrigeration systems, aircraft, power plants, computers, electronics, and radiators^1,2^. Fins primarily facilitate heat dissipation through convection and radiation. For effective application, it is essential to determine the temperature distribution along the fin. When selecting a fin, important factors include its geometry, effectiveness, efficiency, heat dissipation rate, cost-effectiveness, and manufacturing complexity^3^. The performance of fins is impacted by variables such as their length, cross-sectional area, shape, environmental conditions, and the material properties used^4^. Fins are generally categorized into radial, longitudinal, and pin types, with longitudinal fins—featuring a consistent cross-section—being particularly efficient. These types of fins are especially advantageous in natural convection applications, where the heat transfer coefficient is relatively low^5,6^.

Several researchers have investigated ways to improve fin design. Luo et al.^7^ used trapezoidal fins to enhance heat transfer in thermal storage units. Their study analysed temperature distribution, optimal fin size, and the liquid-solid phase diagram, finding that a ratio of the upper to lower base lengths (h_1_/h_2_) of 5 provided optimal heat transfer. Guo et al.^8^ compared the temperature distribution and solid-liquid interface of fractal fins with conventional rectangular fins. Their results showed that fractal fins dissipated more heat due to improved heat conduction, which surpassed natural convection. Sowmya et al.^9^ explored transient temperature distribution in a rod, considering convection, radiation, and internal heat generation. They found a non-linear temperature-time graph that increased over time, suggesting a significant rise in the heat transfer rate. Xu et al.^10^ used rectangular fins in a shell-and-tube heat storage unit to optimize phase-change materials. Their study revealed that the number, positioning, and structure of the fins had a greater impact on performance than the fin’s conductivity.

Lisowski and Lisowski^11^ utilized a 6 × 6 array of longitudinal fins to enhance heat removal from ambient air vaporizers (AAV). Their research focused on pressure and velocity variations based on fin arrangement and pitch values. The results demonstrated a substantial increase in wind load and heat transfer rate with a 45º angle fin arrangement compared to a perpendicular arrangement. Cai et al.^12^ investigated longitudinal fins in a shell and tube ice storage (STIS) unit, analysing how the ice storage ratio and energy discharge ratios were influenced by the position, pitch, and number of fins. Their study revealed that the solidification process is primarily affected by the heat transfer characteristics of the fins and the heat transfer at the solid-liquid interface. Sampath et al.^13^ examined both rectangular and tapered fins, obtaining heat transfer rates and temperature distributions through theoretical and simulation models. The overall heat transfer rates for the rectangular and tapered fins were found to be 12 W and 10 W, respectively. Patel et al.^14^ explored a phase-changing material (PCM) based turbo tube heat storage system (TTLHSS), determining the melting and solidification rates of the PCM. Their results indicated that using four longitudinal fins reduced the melting and solidification times by 60% and 46.15%, respectively. Zhu and Qiu^15^ conducted a study using annular and longitudinal fins to investigate phase-changing materials, focusing on factors like natural convection, fin position, heat transfer fluid, and the melting process. Their findings revealed that the melting time for phase-changing materials with annular fins was consistently 10% shorter than with longitudinal fins. Turkyilmazoglu et al.^16^ analysed longitudinal aluminium fins with a trapezoidal cross-section, examining energy transfer rates and fin efficiency. Their results showed that fin efficiency reached 93%, but declined as the Peclet number increased. Marcinkowski and Taler^17^ calculated the efficiency of fins mounted on pipes, comparing numerical results with experimental data to assess how fin efficiency varied with the heat transfer coefficient. They found that fin efficiency remained at 93% but decreased as the heat transfer coefficient increased. Feng et al.^18^ investigated the efficiency of H-type fins using both experimental and numerical methods, considering geometric parameters such as fin height, thickness, and slit width. Their results showed that fin efficiency improved with greater thickness and thermal conductivity but decreased with higher surface convective coefficients. The experimental and numerical data were consistent. Bošnjaković et al.^19^ performed a similar analysis on annular fins using numerical simulation, predicting performance across Reynolds numbers from 2000 to 18,000. Their results indicated that fin efficiency decreased as the air Reynolds number increased. Huang and Chung^20^ examined the thermal properties of various fin shapes—annular, rectangular, triangular, parabolic, and hyperbolic. They found that fin efficiency increased with higher Biot numbers and relative humidity, while the thermal conductivity of both the tube and the fin had minimal impact on the efficiency of the annular fin.

Sonawane et al.^21^ simulated engine cylinders with fins made from Aluminium alloys 204 and 6061, exploring the effects of fin geometry, thickness, and pitch on heat flux and temperature distribution. Their findings indicated that Aluminium alloy 6061 delivered superior heat transfer performance. Similarly, Subbiah et al.^22^ compared three materials—cast iron, Aluminium alloy 6061, and AZ32 magnesium alloys—using simulations to analyse heat flux and temperature distribution in fins attached to an engine block. They concluded that Aluminium alloy 6061 exhibited the best heat transfer performance due to its high thermal conductivity. Din et al.^23^ compared trapezoidal fins with exponential fins, assessing their efficiency and effectiveness. The study found that exponential fins provided a higher heat transfer rate and outperformed trapezoidal fins. Additionally, fin efficiency improved as thermal conductivity and the radiation-convection number increased. Kaladgi et al.^24^ investigated circular perforated fins, calculating heat flux and temperature gradients using ANSYS Fluent. Their results demonstrated that these fins achieved significant temperature reductions and efficiently dissipated heat. Souida et al.^25^ conducted a numerical study on conical-shaped fins, exploring five different height-to-diameter ratios and varying air Reynolds numbers. They found that as both the height-to-diameter ratio and the Reynolds number increased, thermal resistance decreased, while pressure drop increased. Dixit et al.^26^ examined the thermal performance of a plate pin-fin heat sink with NACA 00XX-shaped pin-fins between parallel plate fins, analyzing the effects of relative thickness and streamwise relative pitch over a Reynolds number range of 300–3300. The maximum thermo-hydraulic performance factor of 1.93 was achieved at the Reynold’s number of 1500 with relative thickness 0.45 and pitch ratio 0.25. Experimental results were validated with the ANSYS simulations, empirical correlations for Nusselt number and friction factor were developed, and machine-learning models were applied, with Gaussian Process Regression demonstrating the highest prediction accuracy (R² > 0.99). Dixit et al.^27^ presented a novel approach which enhanced the heat sink performance by employing square, circular, triangular, and NACA 0040 pin-fin profiles arranged streamwise between parallel plate fins while maintaining a constant blockage area. A systematic parametric analysis was conducted over streamwise pitch ratios of 0.24–0.28, profile thickness ratios of 0.3–0.5, and Reynolds numbers ranging from 300 to 3300. CFD results indicated that the NACA 0040 profile delivers superior thermal performance, which was experimentally validated. Korpyś et al.^28^ presented heat transfer analysis of short-channel structured packing in chemical reactors, evaluating heat transfer coefficients, streamlines, and fluid temperatures. Three modified channel geometries with rounded inlets and altered outlets were compared with a classic straight-wall structure to reduce flow vortices. Results indicated that the modified designs showed significantly enhanced heat transfer. Cui et al.^29^ examined the composite fins integrated into a PCM-based heat storage module for hot-water production in building heating. Numerical and experimental results showed that increasing the length and number of cross fins improved the melting uniformity, leading to a 7.37-fold increase in thermal storage rate and a 781.25% enhancement in temperature response, highlighting the design’s potential for energy-efficient building applications. Das et al.^30^ investigated various cylindrical fin designs to enhance heat transfer by altering fin geometry, material, number, and size. Finite element thermal analysis revealed that the modified design using aluminum alloy 6061 achieved the highest heat flux while maintaining lower weight than cast iron and copper, making it the most efficient material choice. Table 1 gives a summary of the literature on the gin geometry and heat transfer performance.

**Table 1:** Summary of existing literature on fin geometry and heat transfer performance.

Reference number	Authors	Fin geometry/configuration	Operating conditions/parameters	Pictorial/numerical representation	Major quantitative findings
^7^	Luo et al.	Trapezoidal fins	PCM thermal storage; fin size ratio (h₁/h₂)	Temperature contours, phase diagrams	Optimal heat transfer at h₁/h₂ = 5
^8^	Guo et al.	Fractal vs. rectangular fins	Natural convection dominated regime	Solid–liquid interface plots	Fractal fins showed higher heat dissipation than rectangular fins
^9^	Sowmya et al.	Uniform rod (fin analogy)	Transient analysis with convection, radiation, heat generation	Temperature–time plots	Non-linear temperature rise indicating increased heat transfer rate
^10^	Xu et al.	Rectangular longitudinal fins	Shell-and-tube PCM storage	Temperature fields, melting fronts	Fin number, position, and structure more influential than conductivity
^11^	Lisowski & Lisowski	Longitudinal fins (6 × 6 array)	Ambient air vaporizers; fin pitch, orientation	Velocity and pressure contours	45° fin orientation significantly increased heat transfer and wind load
^12^	Cai et al.	Longitudinal fins	Shell-and-tube ice storage; fin pitch and number	Ice growth and temperature contours	Solidification governed by fin heat transfer and interface behavior
^13^	Sampath et al.	Rectangular and tapered fins	Theoretical and numerical analysis	Temperature distribution plots	Heat transfer rates: 12 W (rectangular), 10 W (tapered)
^14^	Patel et al.	Longitudinal fins (4 fins)	PCM-based turbo tube storage	Melting/solidification fronts	Melting and solidification times reduced by 60% and 46.15%
^15^	Zhu & Qiu	Annular vs. longitudinal fins	PCM melting under natural convection	Flow and temperature fields	Annular fins reduced melting time by ~ 10%
^16^	Turkyilmazoglu et al.	Trapezoidal aluminium fins	Varying Peclet number	Efficiency curves	Maximum fin efficiency of 93%; efficiency decreased with Pe
^17^	Marcinkowski & Taler	Pipe-mounted fins	Variable heat transfer coefficient	Numerical–experimental comparison	Fin efficiency ~ 93%, decreasing with higher h
^18^	Feng et al.	H-type fins	Variation in fin height, thickness, slit width	Experimental analysis	Efficiency increased with thickness, decreased with convective coefficient
^19^	Bošnjaković et al.	Annular fins	Reynolds number: 2000–18,000	Velocity and temperature contours	Fin efficiency decreased with increasing Reynolds number
^20^	Huang & Chung	Annular, rectangular, triangular, parabolic fins	Varying Biot number, humidity	Comparative efficiency plots	Efficiency increased with Biot number; conductivity had minimal effect
^21^	Sonawane et al.	Engine cylinder fins	Aluminium alloys 204 and 6061	Heat flux and temperature plots	Al 6061 showed superior heat transfer
^22^	Subbiah et al.	Engine block fins	Cast iron, Al 6061, AZ32 Mg alloy	Temperature contours	Al 6061 yielded highest heat flux
^23^	Din et al.	Trapezoidal vs. exponential fins	Radiation–convection interaction	Efficiency and effectiveness plots	Exponential fins showed higher heat transfer and efficiency
^24^	Kaladgi et al.	Circular perforated fins	Forced convection	Temperature gradient plots	Significant temperature reduction and enhanced heat dissipation
^25^	Souida et al.	Conical fins	Reynolds number, height-to-diameter ratio	Pressure and temperature contours	Thermal resistance decreased; pressure drop increased
^26^	Dixit et al.	Plate pin-fin heat sink (NACA 00XX)	Re = 300–3300; pitch and thickness ratios	Experimental visualizations	Max thermo-hydraulic performance = 1.93 at Re = 1500
^27^	Dixit et al.	Square, circular, triangular, NACA 0040 pin-fins	Constant blockage area	Flow and thermal fields	NACA 0040 showed superior thermal performance

A comprehensive review of the literature indicates that most existing studies primarily focus on circular and rectangular cross-sectional fins, particularly in applications such as heat storage units and engine cylinders. These investigations typically vary parameters including fin position, pitch, and geometric specifications to enhance heat transfer performance. In addition, fins have been widely employed in phase change material (PCM) tanks to study their influence on melting and solidification behaviour. Performance metrics commonly evaluated in these studies include fin efficiency, heat transfer rate, and fin effectiveness. Despite these extensive efforts, there remains a clear need for further research on alternative fin geometries and their impact on thermal performance across diverse thermal management applications.

Despite extensive studies on fin performance, several **research gaps** remain. Most investigations have concentrated on circular and rectangular fins, with limited exploration of alternative geometries such as square, triangular, trapezoidal, or rectangular profile fins. The combined effects of fin geometry, material and orientation on heat transfer performance have not been systematically analyzed in a single study. Moreover, the integration of experimental, theoretical, and simulation approaches for comprehensive evaluation is limited, and aspects such as thermal stress and safety factors are rarely considered alongside conventional heat transfer metrics. In applications involving phase change materials (PCMs), research has largely focused on standard fin shapes, leaving potential performance improvements through innovative geometries largely unexplored. This study seeks to bridge these gaps by offering a thorough analysis of these factors, employing a multi-method approach to improve the understanding and optimization of fin performance.

The primary **objective** of the current study is to comprehensively investigate the thermal performance of short fins with various cross-sectional geometries and materials under natural convection conditions. Specifically, the study aims to evaluate heat transfer rates, fin efficiency, and effectiveness through a combination of experimental measurements, finite element analysis (FEA), and simulations. By systematically comparing multiple geometries and materials, the study seeks to determine optimal fin configurations, validate numerical models against experimental data, and provide practical insights for the design and optimization of efficient thermal management systems.

Motivation: The motivation for conducting experimentation, simulation, and theoretical analysis of fins is to optimize heat transfer performance. Experimental analysis offers real-world validation, ensuring that theoretical and simulation results align with actual performance while revealing practical challenges. Simulations provide a cost-effective way to evaluate various designs and conditions, enabling the optimization of fin performance. Theoretical analysis delivers critical insights into heat transfer processes, establishing a baseline for comparison and informing the design process. Together, these methods form a comprehensive approach to refining fin design and maximizing heat dissipation efficiency.

Strengths: The strengths of the fin research lie in its comprehensive approach, combining experimental, simulation, and theoretical analysis to optimize heat transfer performance. Experimental analysis validates real-world performance, simulations enable cost-effective testing, and theoretical analysis offers foundational insights. The integration of these methods provides comprehensive data on factors affecting fin performance and offers engineers flexibility in optimizing heat transfer and efficiency.

### Originality and contibution towards the heat transfer study

The originality of the present study lies in its integrated and systematic evaluation of short fins with multiple cross-sectional geometries and materials, combining experimental investigations, and finite element analysis (FEA) simulations. Unlike previous studies that focus on a single fin type or material, this work provides a comparative assessment of square, circular, rectangular, trapezoidal, and triangular fins, offering new insights into their thermal performance, efficiency, and effectiveness under identical natural convection conditions. Additionally, the study examines airflow characteristics, turbulence effects, thermal stresses, and safety factors, aspects that are rarely addressed alongside conventional heat transfer metrics. For readers, the study contributes by identifying optimal fin shapes and materials, validating predictive numerical models, and providing practical guidance for designing high-performance heat sinks and thermal management systems, thereby bridging the gap between theoretical analysis and real-world engineering applications.

### Novelty of work

1. Integrates experimental, theoretical, and simulation approaches to analyze heat dissipation in short fins.
2. Introduces a custom experimental setup for evaluating multiple fin geometries under a constant heat source.
3. Provides a unified comparison of fin shapes and materials, including thermal stress, and safety factors.
4. Identifies optimal fin configurations through correlated analysis of geometry, material properties, and thermal performance, with experimental validation of numerical models.

## Methodology

**Fig. 1:**
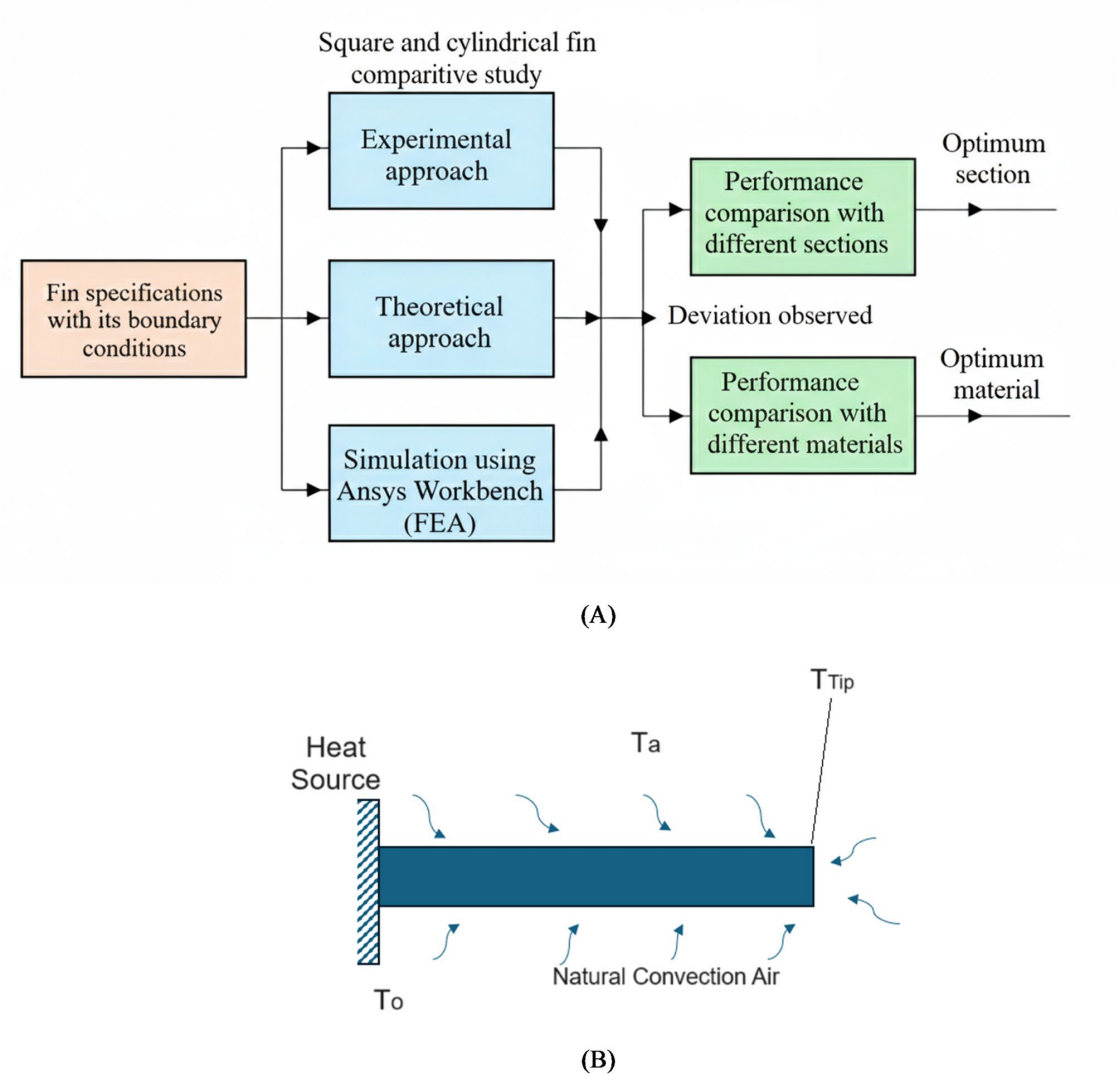
(**A**) Methodology adopted in the study of different cross-sections and materials of the fin. (**B**) Schematic sketch of a short fin.

This study focuses on determining both thermal properties surrounding the fin. Figure 1A provides an overview of the research. By evaluating the input parameters for the fins, various performance metrics for different cross-sectional shapes are assessed through experimental, theoretical, and simulation methods. The input parameters include fin length (0–80 mm), heat input (30 W), ambient air temperature (25 °C), natural air velocity (0.2 to 0.5 m/s), heat transfer coefficient (25–55 W/m²°C), and material properties of the fins. The performance metrics evaluated are temperature distribution along the fin, heat transfer rate, fin efficiency, fin effectiveness, thermal resistance, and thermal stress. The analysis considers multiple fin cross-sectional shapes—square, circular (cylindrical), rectangular, trapezoidal, and triangular—to enable a systematic comparison of their thermal and flow performance under identical operating conditions.

The results are compared, and deviations are analyzed. Additionally, a fluid flow study is conducted to analyze the airflow characteristics around square and round fins. In the end, a theoretical study is conducted by considering different crossection and material fins and the maximum fin performance is evaluated. For the purpose of comparison length and volume of the fin are maintained constant even though there is a change in the cross section area and perimeter.

A short fin with a fixed length and cross-sectional area is attached to a heat source, and various fin parameters are evaluated using theoretical equations. The results are then compared with experimental data and FEA simulations. Figure 1B illustrates the schematic of the short fin.

The temperature at different points along the fin is given by Eq. (1). Here, m represents the fin factor (m^− 1^), h is the heat transfer coefficient (in W/m²°C), k is the thermal conductivity (in W/m°C), l is the length of the fin, and T denotes the temperature at a distance x from the base of the fin.

The rate of heat loss through conduction in a wall or fin can be balanced by the rate of heat transfer through convection from the surface to the surrounding fluid. Heat transfer by the conduction is equal to the heat transfer by the convection as given by Newton in the Eq. (1).The crossection and surface areas are equal^13,31^.

1 $$-k{A}_{c}{\left(\frac{dt}{dx}\right)}_{x=l}=h{A}_{s}\left(t-{t}_{a}\right)$$

The temperature difference $\left(t-{t}_{a}\right)$ is assumed to be *θ* and the equation is rewritten by Eq. (2)^13,31^.

2 $$\frac{d\theta}{dx}=-\frac{h\theta}{k}$$

On solving the fin equation, the value *θ* is determined and presented in the Eq. (3)^13,31^.

3 $$\theta={C}_{1}{e}^{mx}+{C}_{2}{e}^{-mx}$$

*C*_1_ and *C*_2_ are arbitrary constants whose values are to be determined from the boundary conditions at the base and at the tip of the fin.

On solving the equation, temperature of the fin at any point in the fin is determined using Eq. (4). *θ* represents the temperature difference between temperature at any point of the fin and ambient temperature where as *θ*_o_ reperesents the difference between root and ambient temperature. The temperature distribution along the fin is affected by multiple factors, including material properties, fin geometry, boundary conditions, and the characteristics of the surrounding fluid^13^.

4 $$\frac{\theta}{{\theta}_{o}}=\frac{T-{T}_{a}}{{T}_{o}-{T}_{a}}=\frac{Cosh\left(m\left(l-x\right)\right)+\frac{h}{km}sinh\left(m\left(l-x\right)\right)}{\mathrm{cosh}\left(ml\right)+\frac{h}{km}\left(\mathrm{sinh}\left(ml\right)\right)}$$

The heat transfer rate of a fin is given by Eq. (5), where ​ ${\dot{Q}}_{fin}$represents the rate of heat transfer (in watts), A_c_ is the cross-sectional area (in m²), and T_o_​ and T_a_​ are the base and ambient temperatures (in °C), respectively. Heat transfer in short fins occurs through conduction from the base to the tip, with convection removing heat via the surrounding fluid. Due to their limited length, short fins have lower heat transfer efficiency, as heat dissipates over a shorter distance, and the tip dissipates less heat than the base. The effectiveness depends on material properties, fin geometry, boundary conditions, and fluid characteristics^13^.

5 $${\dot{Q}}_{fin}=\sqrt{hpk{A}_{C}}\left({T}_{o}-{T}_{a}\right)\left(\frac{\mathrm{tanh}\left(ml\right)+\frac{h}{km}}{1+\frac{h}{km}\mathrm{t}\mathrm{a}\mathrm{n}\mathrm{h}\left(ml\right)}\right)$$

Fin efficiency is given by Eq. (6) and is defined as the ratio of the heat transfer rate of the fin to the heat transfer rate of an ideal fin. Fin efficiency is expressed as a percentage. Fin efficiency measures how effectively a fin transfers heat from a surface to the surrounding fluid, with higher efficiency indicating better heat dissipation. It is influenced by factors such as geometry, material properties, length, and the temperature difference between the fin and fluid. It is given by percentage^32^.

6 $${\eta}_{fin}=\frac{{\dot{Q}}_{fin}}{hpl({T}_{o}-{T}_{a})}$$

Fin effectiveness is defined as the ratio of the heat transfer rate with the fin to the heat transfer rate without the fin. It is given by Eq. (7). Fins with greater effectiveness offer improved thermal management in various applications. Numerator and denominator terms have a unit of Watts^32^.

7 $$\varepsilon = \frac{{\dot{Q}_{{fin}} }}{{hA_{c} (T_{o} - T_{a} )}}$$

Numerical model to determine the temperature distribution along fin: The finite element method (FEM) for temperature distribution in fins is based on the equation F = K×T which links the force vector (heat flux), the stiffness matrix (thermal conductivity and geometry), and the temperature vector (unknown temperatures at the nodes). The process includes several key steps: Formulating the conductivity and convection Matrix K, Assembling the Global conductivity matrix, Formulating the force vector, Solving the system, and Post-processing, which involves analysing the temperature values. Equation (8) to (13) follow the steps to determine the temperature values at any given point.

By considering the heat transfer from the fin by convection, the matrix method is adopted to obtain various parameters like temperatures at different points. The general heat transfer equation is used for relating the element force vector, conductivity matrix, and temperature matrix. It is given by Eq. (8). The conductivity matrix is given by the sum of the thermal conductivity matrix and convection matrix as given in Eq. (9). Similarly, the element force vector matrix is related to the heat generation vector matrix, heat flux vector matrix, and boundary convection vector^33^.

8 $$\left[F\right]=\left[K\right]\left[T\right]$$

9 $$\left[K\right]=\left[{K}_{c}\right]+\left[{K}_{h}\right]$$

K is the thermal conductivity, A_c_ is the area of cross section, L is the length, h is the heat transfer coefficient and p is the perimeter^33^.

10 $$\left[K\right]=\frac{k{A}_{c}}{L}\left[\begin{array}{cc}1&-1\\-1&1\end{array}\right]+\frac{hpL}{6}\left[\begin{array}{cc}2&1\\1&2\end{array}\right]$$

Element force vector matrix is given by Eq. (11) where individual matrix, heat generation vector matrix, heat flux vector matrix and boundary convection vector are determined.

11 $$\left[F\right]=\left[{F}_{\stackrel{-}{Q}}\right]-\left[{F}_{q}\right]+\left[{F}_{h}\right]$$

Let $\stackrel{-}{Q}$ be the internal heat generation, q be the heat flux, T_a_ is the ambient temperature^33^.

12 $$\left[F\right]=\frac{A\stackrel{-}{Q}L}{2}\left[\begin{array}{c}1\\1\end{array}\right]-\frac{qpL}{2}\left[\begin{array}{c}1\\1\end{array}\right]+\frac{h{T}_{a}pL}{2}\left[\begin{array}{c}1\\1\end{array}\right]$$

Equations (9), (10), (11) and (12) are substituted in the Eq. (8) to obtain Eq. (13) and the temperatures at different points along the short fins are determined^33^.

13 $$\left(\frac{AQL}{2}\left[\begin{array}{c}1\\1\end{array}\right]-\frac{qpL}{2}\left[\begin{array}{c}1\\1\end{array}\right]+\frac{h{T}_{a}pL}{2}\left[\begin{array}{c}1\\1\end{array}\right]\right)=\left(\frac{k{A}_{c}}{L}\left[\begin{array}{cc}1&-1\\-1&1\end{array}\right]+\frac{hpL}{6}\left[\begin{array}{cc}2&1\\1&2\end{array}\right]\right)\left[\begin{array}{c}{T}_{1}\\{T}_{2}\end{array}\right]$$

The matrix obtained in Eq. (13) is used as the governing equation for finite element method software Ansys. Initially, the fins are modelled according to their dimensions using Creo software and analysed using the thermal stress analysis platform in ANSYS Workbench. First, the 3D geometry is modelled using CREO software, considering various shapes such as square, or cylindrical. Next, the geometry is meshed into small elements to balance accuracy with computational efficiency. A hexahedral mesh with an element size of 2 mm is used for the analysis. Boundary conditions, such as fixed support, temperature, air velocity and air convection, are applied. Theoretical, experimental, and simulation results are compared, to determine the deviation among the three methods. If the deviations are within 15% limit, the theoretical equations are employed to determine fin performance metrics such as efficiency, effectiveness, and heat transfer rate for various cross-sections and materials.

## Construction of experimental setup

**Fig. 2:**
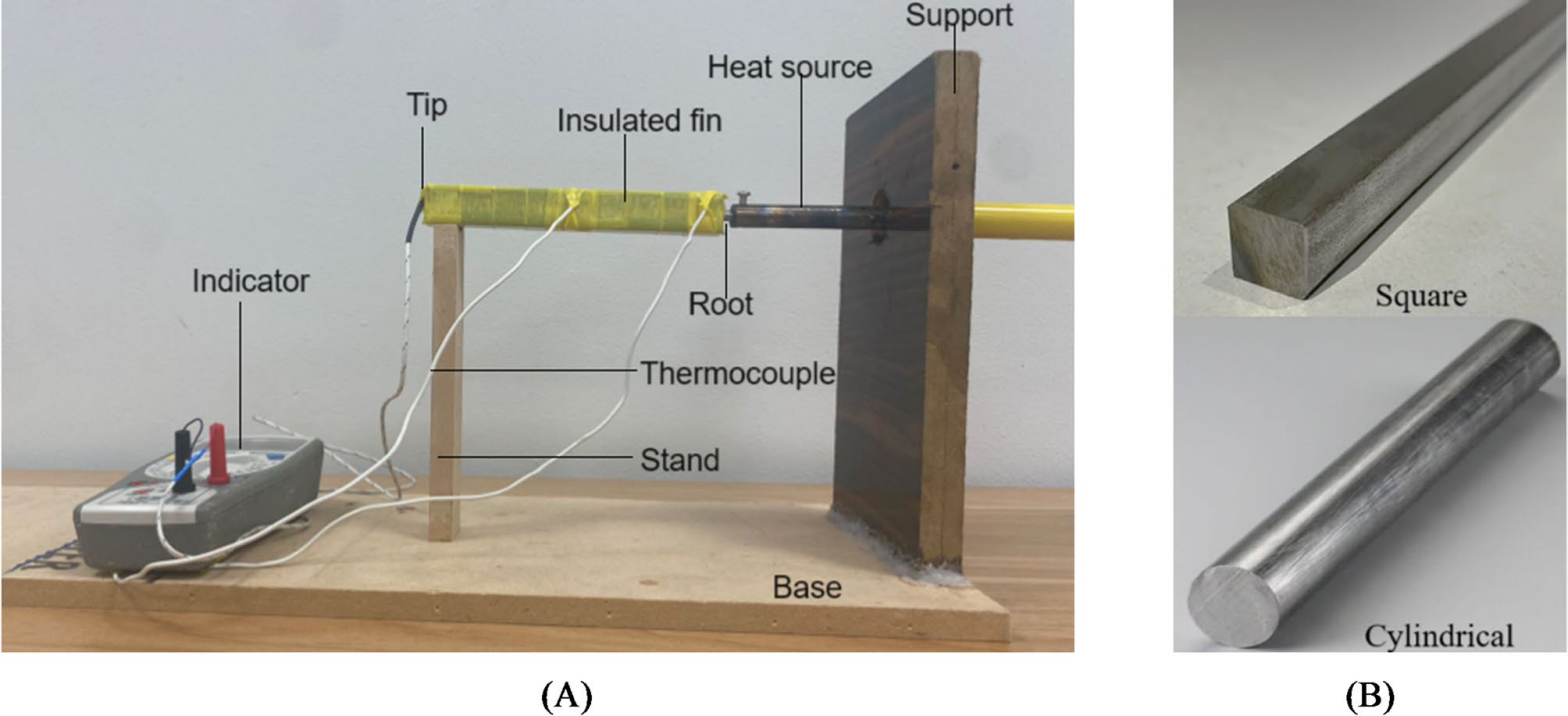
(**A**) Experimental set up. (**B**) Square and cylindrical crossection fins.

The setup consists of a wooden frame that supports fins with various cross-sections and a length L. One end of each fin is heated by a constant heat source, such as a soldering iron, while natural air circulates around the fin. An experiment is conducted by measuring the temperature at various points along the fin, from the base to the tip, using a thermocouple. In the present analysis, Type K (Chromel–Alumel) thermocouples were utilized for temperature measurement owing to their broad operating range, thermal stability, and suitability for heat transfer investigations involving short fins. Before conducting the experiments, all thermocouples were systematically calibrated to ensure measurement accuracy and reliability. The calibration procedure was carried out using a standard temperature calibration setup, wherein the thermocouples were benchmarked against a certified reference thermometer within a controlled temperature bath across the relevant operating range. Calibration curves were generated by recording deviations at multiple temperature points, and appropriate correction factors were applied during data acquisition. This rigorous calibration process ensured consistent, accurate, and repeatable temperature measurements throughout the experimental study. Figure 2A, B illustrate the experimental test rig and the types of fins used in the experimentation, while Table 2 provides details of the components in the entire unit.

**Table 2:** Equipment used in the experimentation.

Sl. No	Equipements used	Purpose	Specification
1.	Fins or extended surface	Test sample	Sqauare crossection: 12.8 mm x 12.8 mm, Length 80 mm, Mild steel. Circular crossection: 14.3 mm diameter and length 80 mm, Mild steel.
2.	Base and frame	Support	Wood
3.	Soldering iron	Heat source	Power: 30 Watts, voltage 220 V, soldering iron length: 241.3 mm, cable length = 1.39 m.
4.	Thermocouple Type K (Chromel–Alumel) thermocouples	Temperature measurement	Temperature range: 0 to 400 °C, probe diameter and length: 5 mm and 150 mm, accuracy: ±0.5% and resolution 0.1 °C.
5.	Clamp meter	Voltage and current	400 A AC, 600 Volts AC/DC voltage, & resistance of 40 MΩ.

### Uncertainity analysis

The uncertainty in performance parameters arising from instrument errors is assessed through a comprehensive error analysis. These uncertainties influence the evaluation of fin performance, with errors in calculating heat transfer rate, efficiency, and effectiveness being quantified using Eq. (5).Let U represent the function, with y_1_,y_2_,…,y_n_​ denoting the intervals of uncertainty and x_1_,x_2_,…,x_n_​ as the independent variables. The total uncertainty W_R​_ is determined using root sum of squares (RSS) as shown in the Eq. (14)^34^.

14 $${\mathrm{w}}_{R} = \left[ {\left( {\frac{{\delta U}}{{\delta X_{1} }}y_{1} } \right)^{2} + \left( {\frac{{\delta U}}{{\delta X_{2} }}y_{2} } \right)^{2} + \left( {\frac{{\delta U}}{{\delta X_{3} }}y_{3} } \right)^{2} + \ldots + \left( {\frac{{\delta U}}{{\delta {\mathrm{X}}}}w_{n} } \right)^{2} } \right]^{{0.5}}$$

Errors in calculating the heat transfer rate, efficiency, and effectiveness of the fins are described by Eq. (5).

$\frac{\partial\left({\dot{Q}}_{fin}\right)}{{\dot{Q}}_{fin}}$, $\frac{\partial\left({\eta}_{fin}\right)}{{\eta}_{fin}}$ and $\frac{{\partial \left( \varepsilon \right)}}{\varepsilon }$ are evaluated considering the sensitivity of the measuring instruments which is shown using the equations (14 A), (14 B) and (14 C).

14A $$\begin{aligned} \frac{{\partial \left( {\dot{Q}_{{fin}} } \right)}}{{\dot{Q}_{{fin}} }} & = \sqrt {\left( {\frac{{\partial A_{C} }}{{A_{C} }}} \right)^{2} + \left( { - \frac{{\partial \Delta T}}{{\Delta T}}} \right)^{2} + \left( {\frac{{\partial l}}{l}} \right)^{2} + \left( { - \frac{{\partial l}}{l}} \right)^{2} } \\ \frac{{\partial \left( {\dot{Q}_{{fin}} } \right)}}{{\dot{Q}_{{fin}} }} & = \sqrt {\left( {\frac{{0.1}}{{163.84}}} \right)^{2} + \left( { - \frac{{0.001}}{{0.1}}} \right)^{2} + \left( {\frac{{0.1}}{{80}}} \right)^{2} + \left( { - \frac{{0.1}}{{80}}} \right)^{2} } = {\mathrm{1}}{\text{.02\% }}{\mathrm{.}} \\ \end{aligned}$$

14B $$\begin{aligned} \frac{{\partial \left( {\eta _{{fin}} } \right)}}{{\eta _{{fin}} }} & = \sqrt {\left( {\frac{{\partial \dot{Q}_{{fin}} }}{{\dot{Q}_{{fin}} }}} \right)^{2} + \left( { - \frac{{\partial l}}{l}} \right)^{2} + \left( { - \frac{{\partial \Delta T}}{{\Delta T}}} \right)^{2} } \\ & = \sqrt {\left( {\frac{{1.02}}{{100}}} \right)^{2} + \left( { - \frac{{0.1}}{{80}}} \right)^{2} + \left( { - \frac{{0.1}}{{80}}} \right)^{2} } {\text{ = 1}}{\text{.03\% }}{\mathrm{.}} \\ \end{aligned}$$

14C $$\begin{aligned} \frac{{\partial \left( \varepsilon \right)}}{\varepsilon } & = \sqrt {\left( {\frac{{\partial \dot{Q}_{{fin}} }}{{\dot{Q}_{{fin}} }}} \right)^{2} + \left( { - \frac{{\partial A_{c} }}{{A_{c} }}} \right)^{2} + \left( { - \frac{{\partial \Delta T}}{{\Delta T}}} \right)^{2} } \\ & = \sqrt {\left( {\frac{{1.02}}{{100}}} \right)^{2} + \left( { - \frac{{0.1}}{{163.84}}} \right)^{2} + \left( { - \frac{{0.001}}{{0.1}}} \right)^{2} } = {\mathrm{1}}{\text{.42\% }}{\mathrm{.}} \\ \end{aligned}$$

Measurement errors for various performance parameters are detailed in Table 3. With error percentages below 2%, the experimental values are within an acceptable range. Table 4 represents the details of the experimental conditions.

**Table 3:** Error values of the dependent variables.

S. no	Variable	Percentage uncertainty
1.	Heat transfer rate	1.02
2.	Fin efficiency	1.03
3	Fin effectiveness	1.42
4	Temperature	1.50

**Table 4:** Experimental conditions.

S. No.	Material used by the fin, its cross sections and specification of square and circular fins	Specification of fin and experimental parameters	Output parameter	Remarks
1.	Other crossection: Square, Circular Rectangle, Trepezoidal and Triangular Other fin materials: Mild steel Stainless steel, Cast Iron and Titanium. **Square crosssection**: 12.8 mm x 12.8 mm, Length 80 mm, Mild steel. **Circular crossection**: 14.3 mm diameter and length 80 mm, Mild steel.	Length of the specimen: 0 to 80 mm. Area of cross section = 163.84 mm^2^ Temperature range: 77 to 50 °C. Heat Source: 30 W. Natural convection air with ambient temperature: 25 °C. Natural air velocity:0.2 to 0.5 m/s^35^. Root temperature:77 °C. Heat transfer coefficient variation: 25 W/m^2^ºC.	Temperature distribution, Heat transfer rate (W), Fin efficiency (%), Fin factor (m^− 1^), Fin effectiveness, Heat flux (W/mm^2^). Thermal stress (MPa) Deformation due to the heat (mm) Factor of safety (FOS), velocity of air at the fin surface.	The fin temperature decreases along its length, while increasing heat transfer coefficient reduces fin efficiency and effectiveness. Among all geometries, the rectangular cross-section fin exhibits the highest heat transfer rate and overall thermal performance.

## Results and discussions

The current study examines various parameters of square and rectangular fins heated at one end, focusing on the following aspects: (4.1) Temperature distribution along the fin’s length and its validation, (4.2) Determination of the fin’s output parameters (4.3) Comparison of results across different fin cross-sectional shapes (4.4) Comparison of results with fins made from different materials (4.5) Thermal resistance and drop in the pressure.

### Temperature distribution along the fin’s length and its validation

Due to the heat source applied at one end, temperatures along the fin are experimentally measured using a thermocouple. These experimental temperature values are compared with theoretical values obtained from Eq. (1) and results from the finite element method as shown in the Fig. 4.

The temperature along the fin progressively decreases from the base to the tip due to the combined effects of conduction and convection. Heat is conducted through the mild steel fin material, which possesses good thermal conductivity, allowing energy to be efficiently transmitted along the metal fibers from the heated base toward the tip. Simultaneously, natural convection occurs as the surrounding air moves over the fin surface, carrying away heat by replacing the warmer air adjacent to the fin with cooler ambient air, thereby maintaining a temperature gradient that drives convective heat transfer. The surface area of the fin plays a crucial role in this process, as a larger area provides more interface for energy exchange, including minor contributions from radiation, which further enhances heat dissipation to the surrounding environment. However, as the distance from the base increases, the thermal resistance along the fin length accumulates, reducing the temperature gradient and thus diminishing the local heat transfer rate. At the fin tip, this effect is most pronounced: the temperature difference between the fin tip and ambient air becomes smaller, resulting in a lower convective heat transfer rate. Consequently, without additional heat input along the fin, the temperature naturally decreases toward the tip, reflecting the continuous loss of energy through conduction along the fin and convection into the surrounding medium. This interplay between material conduction, convective removal, surface area, and thermal resistance governs the fin’s overall thermal performance and highlights why optimal fin design must consider both geometry and material properties to maximize heat dissipation.

**Fig. 3:**
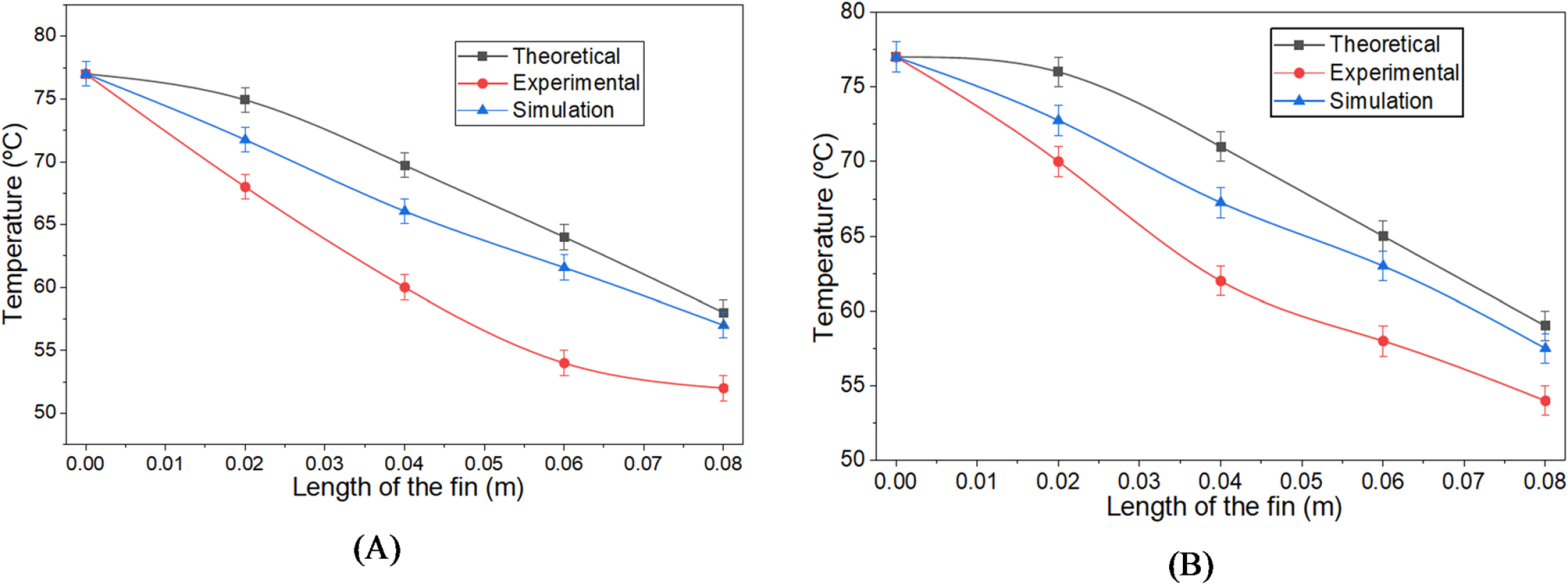
(**A**) Temperature variation along the length of the square cross-section fin. (**B**) Temperature variation along the length of the circular cross-section fin.

Figure 3A, B illustrate the temperature distribution along the fin using experimental, theoretical, and simulation methods for both square and circular cross-section fins. For the square fin, the temperature decreases by 24.67% in the theoretical case, 25.97% in the simulation case, and 32.46% experimentally. Similarly, for the circular fin, the temperature drops by 23.37% in the theoretical case, 25.32% in the simulation case, and 29.87% experimentally.

The deviation between the experimental and theoretical results is 11.53% for the square cross-section and 9.25% for the circular cross-section. In comparison, the deviation between the experimental and simulation results is 5.66% for the square cross-section and 5.45% for the circular cross-section. These discrepancies are attributed to factors such as variations in natural convection air temperature, humidity, mesh size of the body in FEA and air velocity. When compared with the findings of Kannojiya et al.^36^, Hemanth^37,38^, Daliran and Ajabshirchi^39^ and Zhang et al.^40^ which also reported similar analyses, the deviations between experimental and theoretical values fall within a 15% range. Thus, the current results are consistent with the literature.

**Fig. 4:**
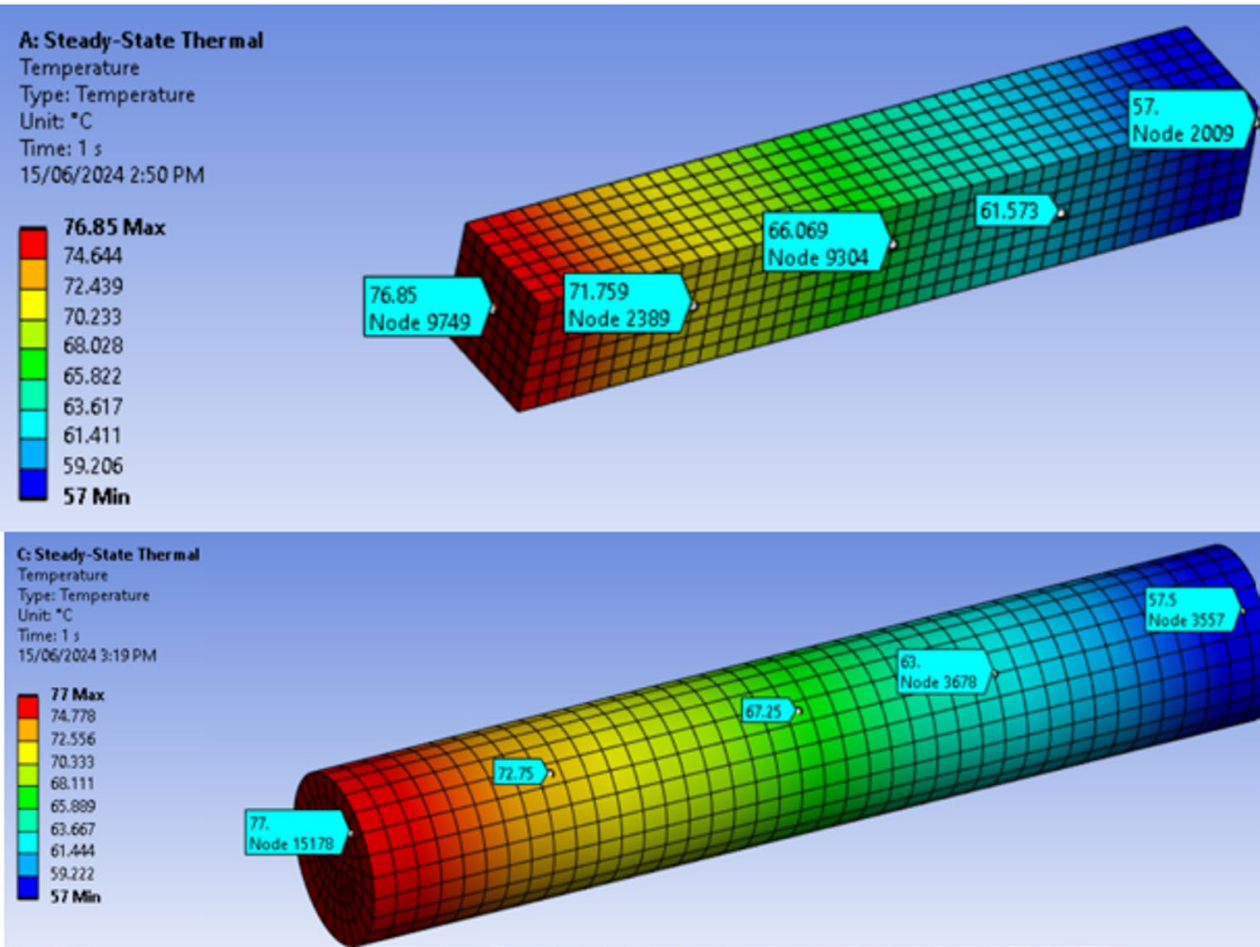
Temperature distribution on square and circular cross-section fins.

The FEA simulation provided intermediate results. Figure 4 shows the temperature distribution along square and circular fins using FEA. It is observed that the temperature at the center and end of the square fin is slightly lower compared to the cylindrical fin, indicating that the square fin transfers heat more effectively than the cylindrical fin.

### Determination of fin output parameters

**Fig. 5:**
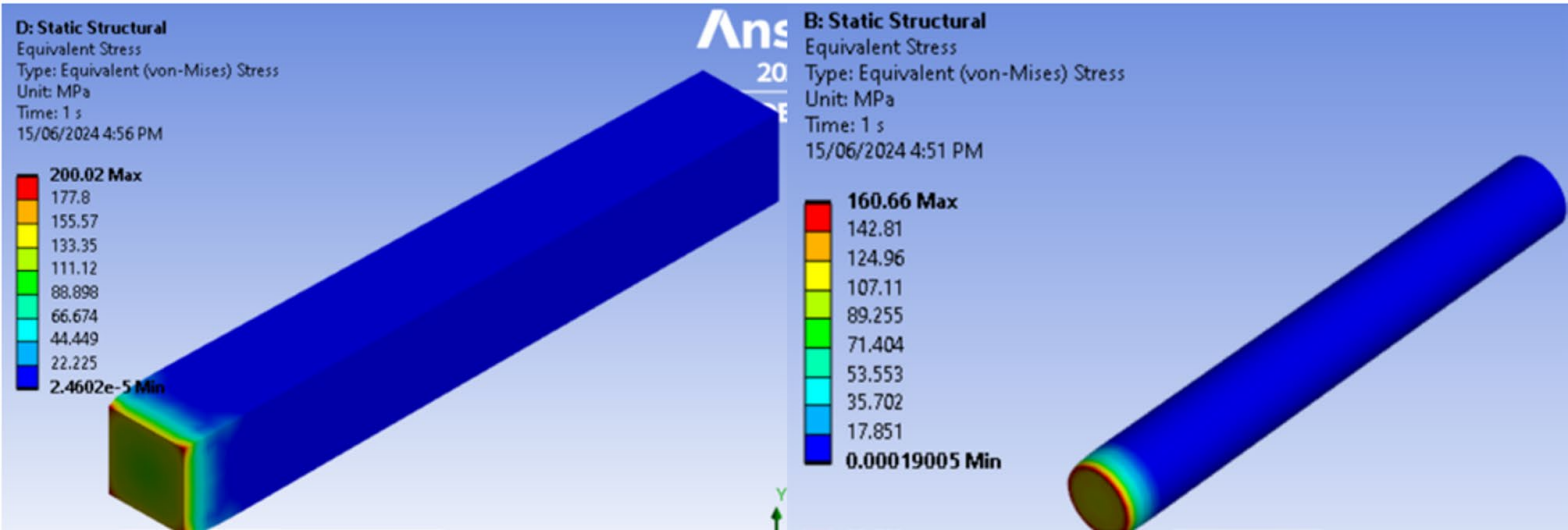
Thermal stress in the square and cylindrical crossection fins.

Considering the fin specifications, temperature distribution at various points along the fin, and natural convection with air, a thermal stress analysis is performed using the FEA tool Ansys Workbench. This analysis determines parameters such as heat flux, deformation, and stresses induced by the heat source. It is found that maximum thermal stress occurs in the square cross-section fins due to stress concentration at the corners. In contrast, the round cross-section fins, which lack such stress concentration, exhibit a higher factor of safety (FOS). The highest stress is observed at the fixed end of the fin where the heat is applied, as shown in Fig. 5.

Square fins experience higher thermal stress than cylindrical fins primarily due to their geometry and the resulting patterns of heat distribution. The sharp corners and edges of square fins act as regions of stress concentration, where abrupt changes in heat flux lead to localized expansion or contraction of the material, creating internal thermal stresses. In addition, the temperature gradients within square fins are often uneven and directional, with heat preferentially flowing along certain paths from the base toward the tip, which further amplifies localized stresses at the corners and edges. In contrast, cylindrical fins possess a smooth, continuous curved surface, allowing heat to distribute more gradually along the radial and axial directions. This more uniform temperature profile minimizes abrupt thermal expansion, resulting in lower internal stresses. Although square fins can offer a slightly larger surface area for convective heat transfer, the geometric discontinuities make them more prone to mechanical strain and thermal fatigue, particularly under repeated heating and cooling cycles. Thus, while square fins may provide enhanced heat dissipation in some cases, their design inherently leads to higher thermal stress compared to the more uniform and mechanically robust cylindrical fins, highlighting the trade-off between thermal performance and structural reliability in fin design.

Using Eqs. (2), (3), and (4), the heat transfer rate, fin efficiency, and fin effectiveness are calculated. The performance parameters for different cross-section fins are summarized in Table 5. The results indicate that factors such as increased surface area, ease of manufacturing, and fluid dynamics around the fin make square fins more efficient compared to cylindrical fins.

**Table 5:** Experimental results of square and cylindrical cross-section fins.

Sl. No.	Parameters	Square fin	Cylindrical fin	Percentage change in the values
1.	Heat transfer rate (W)	5.32	4.66	14.16
2.	Fin efficiency (%)	94.86	93.12	1.8
3.	Fin factor (m^− 1^)	13.17	12.45	5.7
4.	Fin effectiveness	23.71	22.43	5.7
5.	Heat flux (W/mm^2^)	0.018	0.015	20
6.	Thermal stress (MPa)	200.02	160.66	2.44
7.	Deformation due to the heat (mm)	0.043	0.044	2.3
8.	Factor of safety (FOS)	1.24	1.55	25

The percentage deviations obtained while determining the thermal parameters of the fin were compared with the literature from Chakraborty and Sirkar^40^, Mirapalli and Kishore^41^, Mokheimer^42^, and Hussein et al.^43^. It was found that the variations between the two different cross sections fall within a 15% difference. The square fin, with its larger surface area, enhances heat transfer efficiency, thereby improving thermal performance. Additionally, the square edges promote effective airflow, reducing pressure drop and increasing the heat transfer rate. Consequently, the performance of the square fin is slightly superior to that of the cylindrical fin.

#### Theoretical variation of the fin efficiency and effectiveness with the heat transfer coefficient or air velocity

Fin efficiency is defined as the ratio of the actual heat transfer rate of the fin to that of an ideal fin. The convective heat transfer coefficient indicates how effectively heat is transferred between the fin and its surroundings. Fin efficiency and effectiveness decrease as the heat transfer coefficient increases. Higher heat transfer coefficients reduce the temperature gradient along the fin, thereby diminishing its heat transfer capacity and efficiency. Furthermore, as heat is conducted efficiently from the base toward the surrounding air, the temperature gradient along the fin decreases, which reduces the driving force for further heat transfer and consequently diminishes the fin’s thermal effectiveness. In other words, once the fin has dissipated a substantial portion of the heat to the environment, the remaining temperature difference between the fin surface and the ambient air becomes smaller, limiting additional convective transfer. As the air velocity increases, the convective heat transfer coefficient rises, enhancing the overall heat transfer rate. However, higher air velocity also reduces the residence time of air in contact with the fin surface, which can slightly reduce the local heat extraction efficiency per unit area. The geometry of the fin plays a significant role in this context: square fins, with their flat faces and sharp edges, create higher resistance to airflow, inducing local recirculation and longer residence times of air near the fin surface. This effect increases convective interaction and enhances the fin efficiency compared to cylindrical fins, where the smooth, streamlined surface allows air to pass more readily, reducing the intensity of localized heat transfer. Thus, the combination of geometric resistance and airflow dynamics explains why square fins can achieve higher efficiency under the same convective conditions, despite potential trade-offs such as increased thermal stress.

**Fig. 6:**
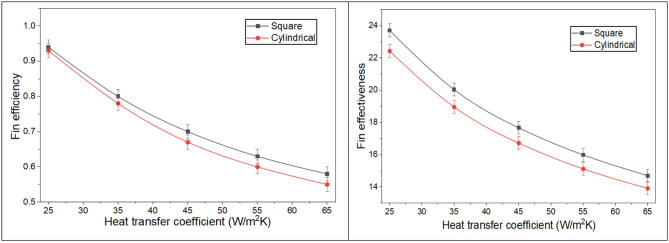
Fin efficiency and effectiveness with the heat transfer coefficient.

Additionally, an increase in the ideal fin’s convective heat transfer rate leads to a reduction in overall efficiency. Fin effectiveness is defined as the ratio of the heat transfer rate with the fin to the rate without the fin. At higher air flow rates, the boundary layer around the fin becomes thinner due to increased turbulence. This turbulence can cause flow separation and reduce fin effectiveness. It has been observed that as the heat transfer coefficient increases, the fin efficiency and effectiveness decrease, with reductions of 38.29% and 38%, respectively, for square cross-section fins, as shown in Fig. 6.

### Comparison of the results with the different shape fins cross-section

**Fig. 7:**
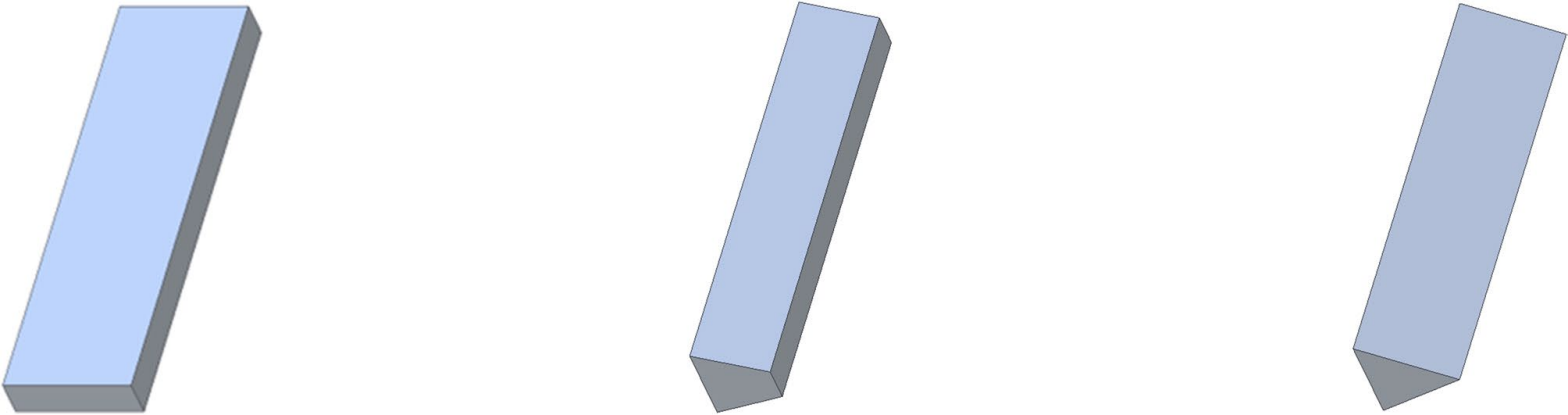
Rectangular, trapezoidal and triangular fins.

Figure 3A and B reveal that the theoretical, experimental, and simulation results show a consistent temperature distribution with minimal deviations. This indicates that theoretical equations can accurately predict fin performance parameters. To further evaluate fin performance, theoretical values for square and cylindrical fins are compared with those of rectangular, trapezoidal, and triangular fins by calculating fin efficiency, effectiveness, and heat transfer rate using Eqs. (2), (3), and (4). Figure 7 presents a 3D sketch of the rectangular, trapezoidal, and triangular fins. In the comparison of these five different cross-section fins, the volume of each fin is kept constant while varying the perimeter. This variation in perimeter leads to differences in efficiency, effectiveness, and heat transfer rate.

#### Fin efficiency with varying cross section

**Fig. 8:**
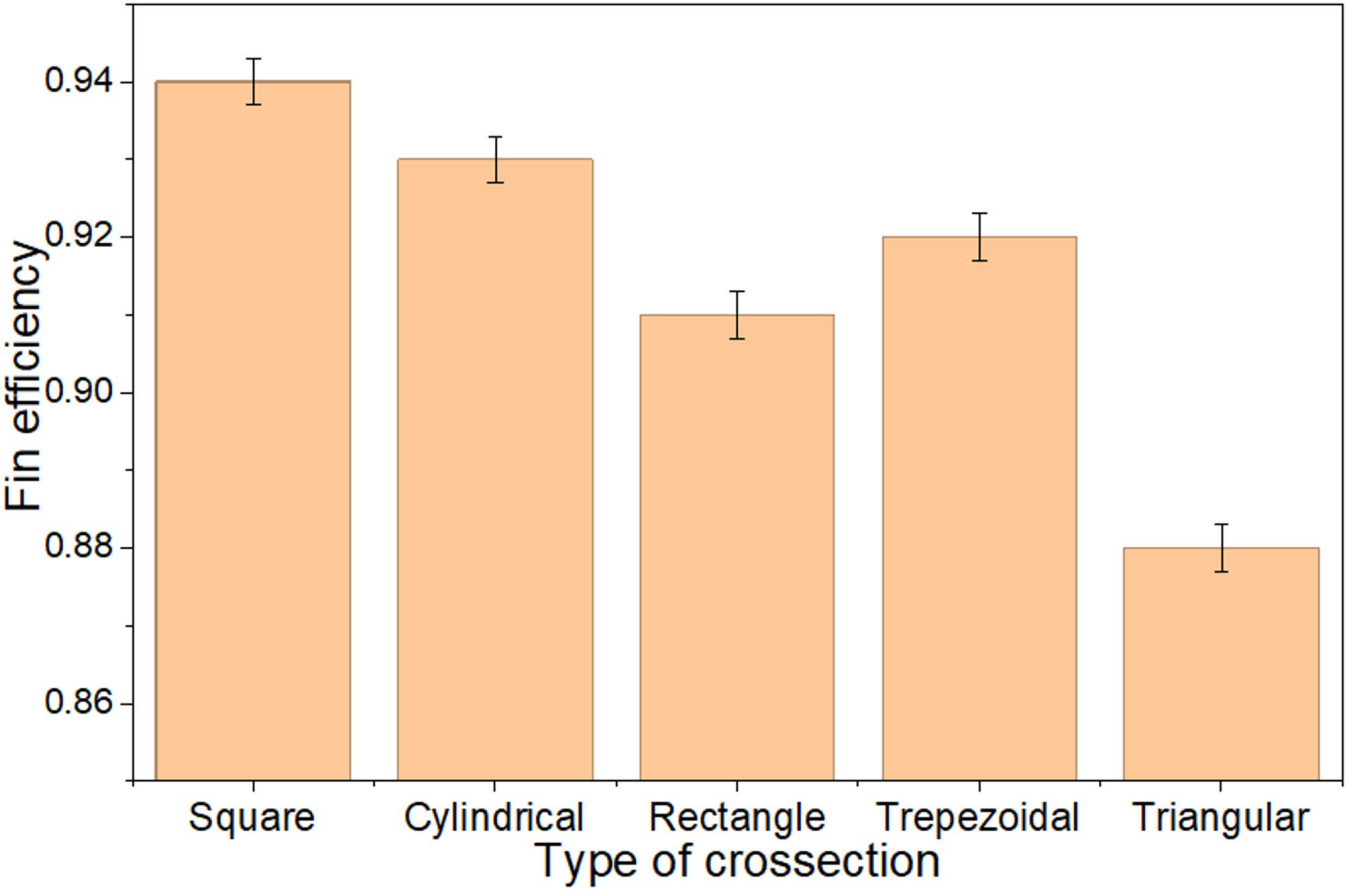
Fin efficiency with the varying cross section.

The perimeters of the square, cylindrical, rectangular, trapezoidal, and triangular fins are 51.2 mm, 44.9 mm, 60 mm, 52.4 mm, and 58.24 mm, respectively, with each fin having a length of 80 mm. Fin efficiency is defined as the ratio of the heat transferred by the fin to the heat transferred by an ideal fin through convection. For the rectangular and square fins, a significant gap is observed between the heat transferred by the fin and the ideal heat transfer, attributed to the higher perimeter which results in a larger surface area. This increased surface area allows for greater heat dissipation by convection. However, the development of the boundary layer around the fin is influenced by the perimeter; a larger perimeter thickens the boundary layer, which reduces convective heat transfer efficiency. The efficiencies of the square, cylindrical, rectangular, trapezoidal, and triangular fins are found to be 94%, 93%, 91%, 92%, and 88%, respectively. The square fin is the most efficient, while the triangular fin shows an 8% lower efficiency. Figure 8 illustrates the fin efficiency across various cross-sections.

#### Fin effectiveness and heat transfer rate with varying cross section

**Fig. 9:**
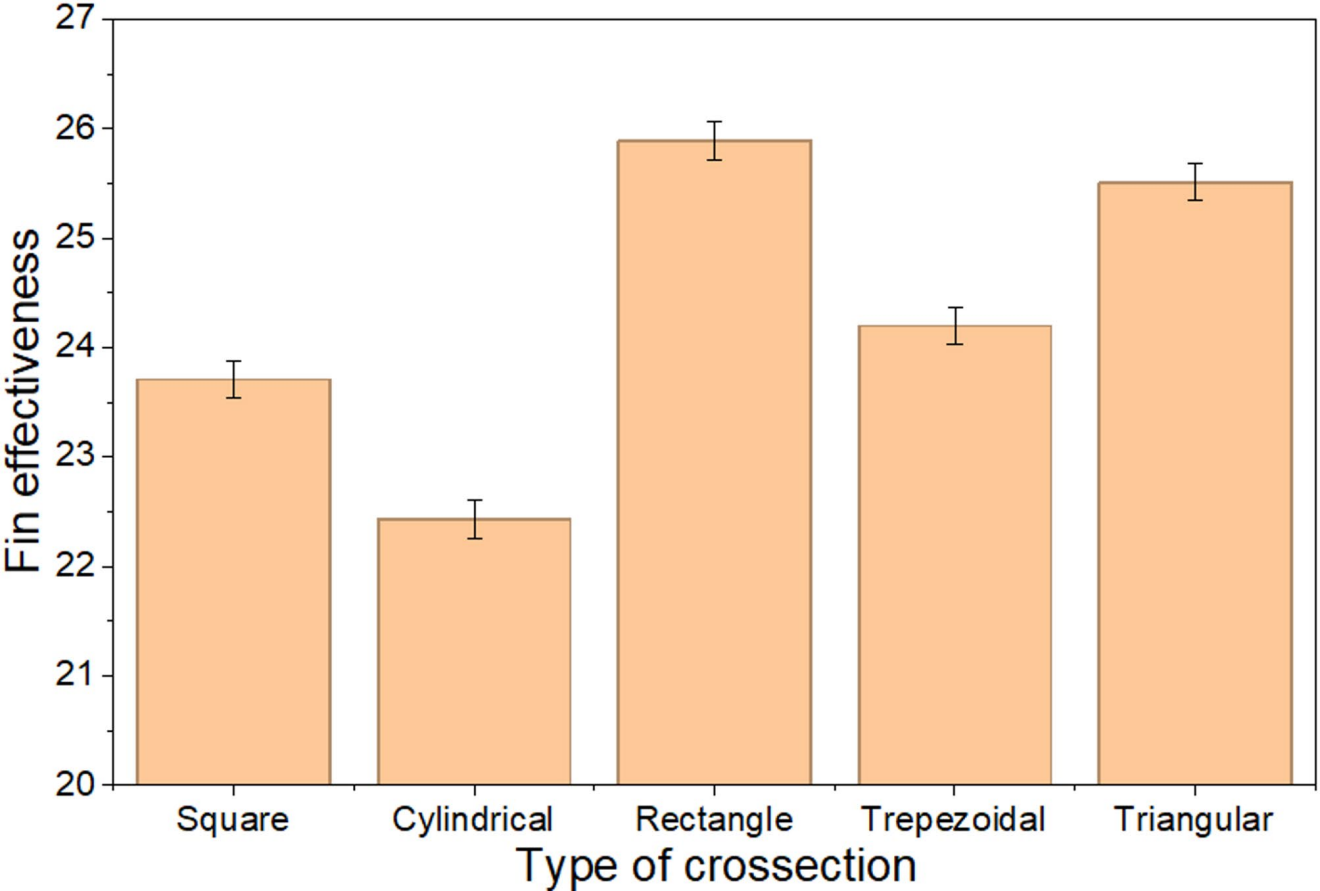
Fin effectiveness with the varying cross section.

**Fig. 10:**
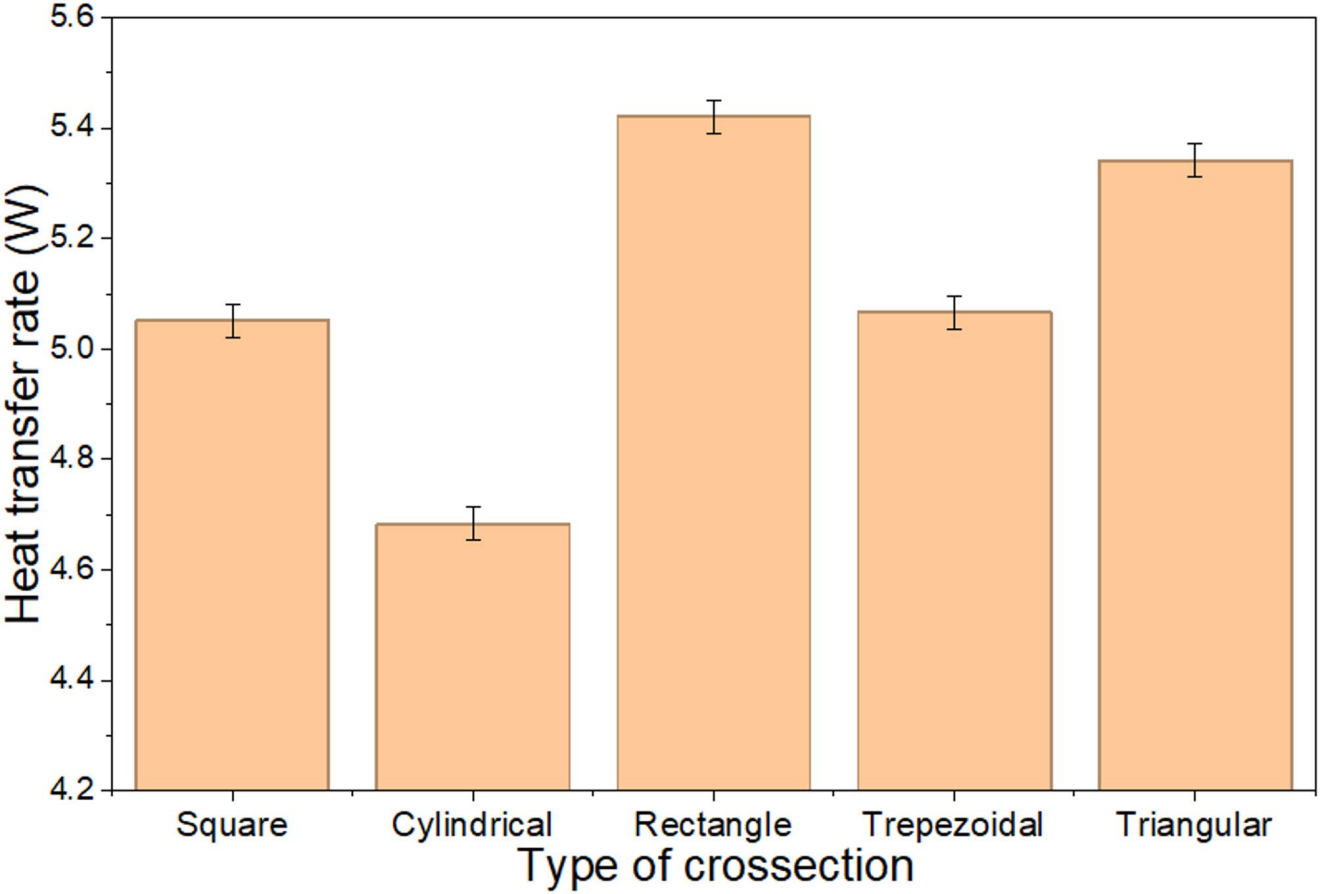
Heat transfer rate with the varying cross section.

Figures 9 and 10 show the variation in fin effectiveness and heat transfer rate. As the perimeter of the fin increases, the heat transfer rate also rises due to the larger surface area, which allows for greater energy exchange with the surrounding fluid. With a larger surface area for heat dissipation, they enable more efficient heat exchange. Their design promotes uniform heat distribution, reducing thermal resistance and improving overall heat transfer.The increased perimeter enhances conduction, thereby improving the overall thermal performance. The data reveal that the rectangular fin achieved the highest effectiveness and heat transfer rate among all the fins with different cross-sections. Specifically, the rectangular fin demonstrated an effectiveness of 25.89 and a heat transfer rate of 5.42 watts, whereas the triangular fin exhibited the lowest performance. The triangular fin’s limited effectiveness is attributed to its smaller surface area, reduced convective heat transfer, uneven flow distribution, and structural instability, which restrict its practical applications.

Fin effectiveness primarily depends on the ratio of the total fin surface area to the base cross-sectional area; therefore, a large surface area ratio combined with a low convective heat transfer coefficient ($h$) leads to a high effectiveness value. Under natural convection conditions, where the heat transfer from the bare base surface is relatively small, an effectiveness in the range of 23–25 is physically reasonable, indicating that the fin dissipates approximately 23–25 times more heat than the unfinned base area alone.

If the total heat input is 30 W while only 4.6–5.4 W is reported as dissipated through the short fins, this indicates that the fins are removing only about 15–18% of the supplied heat. Scientifically, this can occur because heat dissipation in a finned system under natural convection is distributed among multiple pathways: convection from the fin surfaces, convection from the unfinned base plate, thermal radiation to the surroundings, and possible conductive losses through support or mounting structures. Short fins generally have limited surface area and may operate with reduced fin efficiency due to temperature drop along their length, which limits their individual heat removal capacity. At steady state, the total heat rejected by all mechanisms combined must balance the 30 W input. Therefore, the 4.6–5.4 W likely represents only the convective heat transfer from the short fins themselves, not the total heat dissipated by the entire assembly.

### Comparison of the results with different materials of the fin

Fin efficiency, effectiveness, and heat transfer rate were assessed for different cross-sectional materials, including mild steel, stainless steel, cast iron, and titanium, with thermal conductivities of 45 W/mºC, 15 W/mºC, 26.8 W/mºC, and 11.4 W/mºC, respectively. The results indicate that mild steel, with the highest thermal conductivity, delivered the best performance, followed by cast iron, stainless steel, and titanium. Higher thermal conductivity enhances heat transfer from the root to the tip of the fin with lower thermal resistance. High thermal conductivity is associated with a greater atomic packing density, enabling efficient heat transfer through free electrons or phonons. At the atomic level, vibrations occur as heat energy is absorbed at one end and converted into kinetic energy, causing atoms to vibrate and collide with their neighbours. This interaction excites adjacent atoms, prompting them to vibrate as well. Consequently, materials with higher conductivity facilitate more effective.

With high thermal conductivity, mild steel enables rapid heat transfer from the base to the surrounding fluid, enhancing heat dissipation. Additionally, its durability and resistance to thermal stresses further contribute to the fin’s overall efficiency. These material characteristics lead to improved heat transfer performance, greater fin efficiency, and enhanced effectiveness in optimizing heat exchange.

**Fig. 11:**
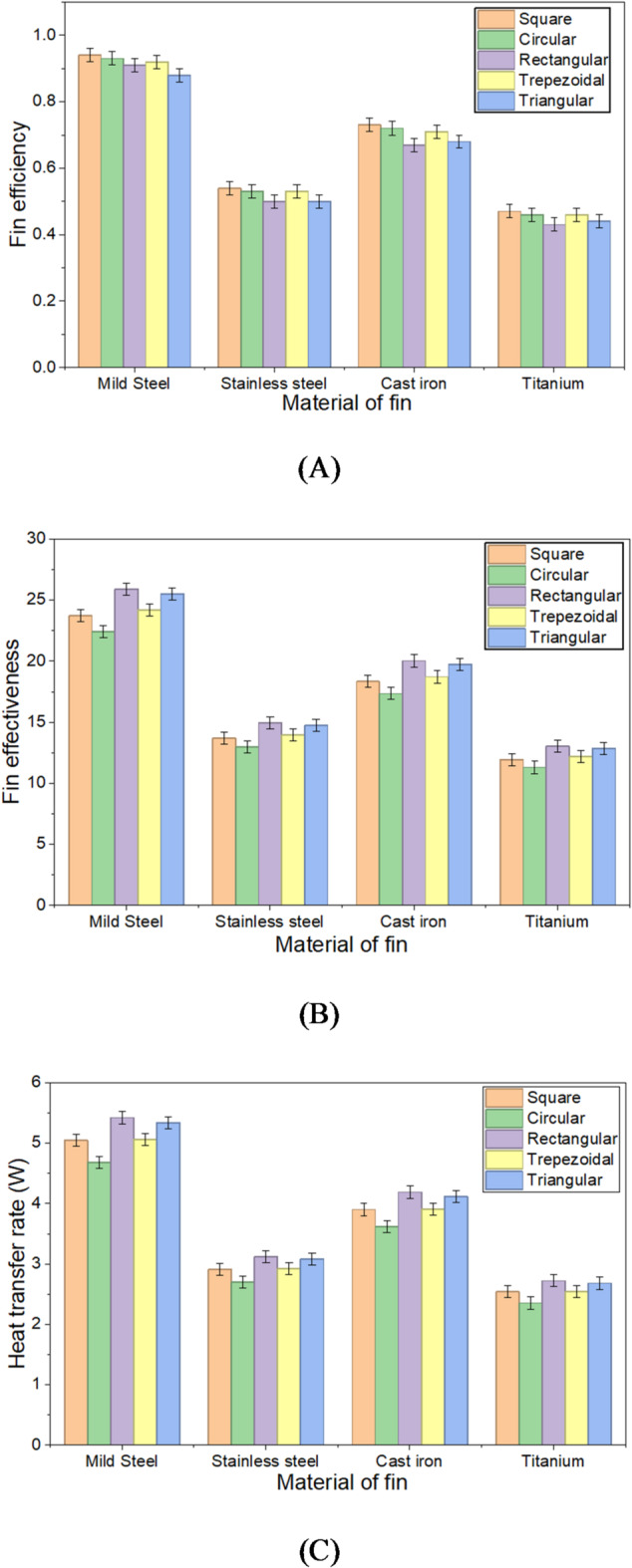
(**A**) Fin efficiency with the varying materials. (**B**) Fin effectiveness with the varying materials. (**C**) Heat transfer rate with the varying materials.

Figure 11A, B, and C illustrate that mild steel consistently outperforms other materials in terms of efficiency, effectiveness, and heat transfer rate. Titanium, in comparison, showed a decrease in fin efficiency, effectiveness, and heat transfer rate by 50%, 49.68%, and 49.70%, respectively.

### Thermal resistance and pressure drop

**Fig. 12:**
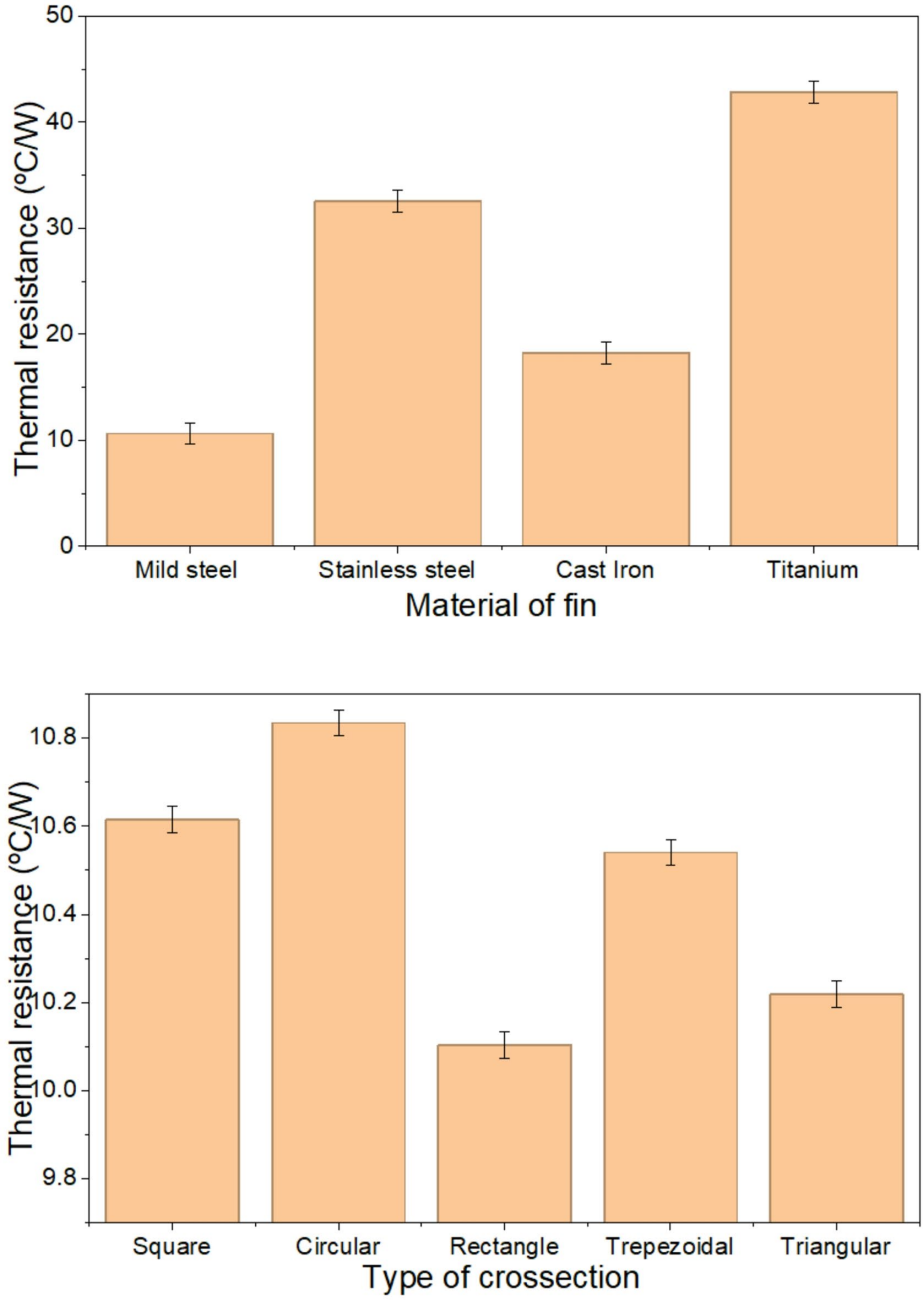
Thermal resistance variation with the material and the type of cross section.

Figure 12 illustrates the variation of thermal resistance across different materials and sections of a fin. Thermal resistance measures a material’s ability to resist heat flow and plays a crucial role in various modes of heat transfer. It is influenced by factors such as length, cross-sectional area, and thermal conductivity. For mild steel, stainless steel, cast iron, and titanium, the thermal resistances calculated were 10.61, 32.55, 18.21, and 42.83 °C/W, respectively, corresponding to thermal conductivities of 46, 15, 26.8, and 11.4 W/mºC for a square cross section fin. This data shows that as thermal conductivity increases, thermal resistance decreases, highlighting their inverse relationship and resulting in a lower temperature drop. In this case, mild steel exhibited the lowest thermal resistance, indicating high heat transfer efficiency.

The thermal resistance of a circular titanium fin is high due to several factors. Firstly, titanium has lower thermal conductivity compared to materials like copper or aluminium, which increases thermal resistance and reduces heat transfer efficiency. Secondly, circular fins have less surface area for heat dissipation compared to more complex geometries, such as rectangular or flat fins, leading to lower heat transfer and higher thermal resistance. Lastly, uneven heat distribution along the titanium surface can cause localized hot spots, further increase thermal resistance and reducing overall heat dissipation. Figure 12 also examines the variation in thermal resistance across different fin cross sections. Five shapes were analyzed: square, circular, rectangular, trapezoidal, and triangular. The thermal resistances for these sections were measured at 10.61, 10.83, 10.01, 10.54, and 10.07 °C/W, respectively. As the cross-sectional area increased, thermal resistance decreased significantly, leading to a reduction in temperature drop. Notably, the rectangular and triangular fins exhibited thermal resistances that were 4.8% and 3.77% lower, respectively, compared to the square fins, enhancing overall heat transfer performance.

#### Pressure drop

In the free convection case of fin, the drop in the pressure is not a primary concern as the natural convection takes place by buoyancy forces and temperature gradients. In the direction of the flow, pressure drop reduces negligibly but it is not referred as a pressure drop.

## Comparison with the literature

The results from the current experiments were compared with those from various studies, as outlined in Table 6. Fin efficiency was chosen as the comparative parameter, as it is independent of the fin’s specifications, ensuring its value remains consistent despite dimensional variations. The comparison demonstrated that the fin efficiency observed in the present study is in line with, and slightly exceeds, the values reported in existing literature, thereby validating the results.

**Table 6:** Comparison of present results with that of the literature values.

Sl. No	Author’s name	Type of fins	Experimental conditions	Fin efficiency
1.	Present study	Square and circular	Length = 0–0.8 m Inlet temperature=77 °C	94.86% & 93.12%
2.	Marcinkowski et al.^17^	Circular	L = 10 mm, Base temperature= 90 °C, heat transfer coefficient variation = 0 to 300 W/m^2^k.	93%
3.	Feng et al.^18^	H-type finned tube	heat transfer coefficient variation = 30 to 70 W/m^2^k. ml varied from 0.25 to 2.5.	73%
4.	Bošnjaković et al.^19^	Annular type	Reynolds number: 2000 to 16,000	78%
5.	Huang and Chung^20^	Annular fin	Radius = 0.2-1	78%
6.	Din et al.^23^	Trapezoidal fins	Convection conduction number N_c_	65%

The present study with respect to its volume and mass of the heat sink, is compared with that of the fin present in the literature. D/L ratio for square, cylindrical and rectangular fin is considered in this study and it yielded the fin efficiency of 94.86%, 93.12% and 86% as shown in the Table 7. Souida et al.^25^ considered conical and cylindrical fin with a D/L ratio of 0.167 and 0.333 which yielded an efficiency of 140% and 130% respectively. It is inferred that with the rise in the D/L ratio efficiency reduced significantly in the current work and that of the literature.

**Table 7:** Comparison of present volume and mass of the heat sink with the literature values.

Sl. No	Author’s name	Type of fins	Experimental conditions	Fin efficiency	Remarks
1.	Present study	Square, Cylindrical and rectangular fin	D/L ratio of 0.16, 0.17 and 0.28	94.86%, 93.12% and 86%	With the rise in the D/L ratio the fin efficiency drops
2.	Souida et al.^25^	Multiple conical and cylindrical fins	D/L ratio of 0.167, 0.333	140%, 130%.	Drop in the fin efficiency with the D/L ratio

## Conclusions

The study offers a comparative analysis of temperature distribution using experimental, simulation, and theoretical approaches, investigates airflow characteristics of square and circular fins, and assesses thermal stress, safety factors, and heat transfer performance across different fin shapes and materials. Based on the results from these various tests and simulations, the following conclusions have been drawn:

1. The average temperature reduction for square and circular cross-section fins is 27.7% and 26.1%, respectively. The deviation between experimental and theoretical results is 11.53% for square fins and 9.25% for cylindrical fins because of conditions of air and its air velocity.
2. The findings showed that the heat transfer rate, fin efficiency, and effectiveness for square fins were 5.32 W, 94.86%, and 23.77, respectively, while for cylindrical fins, these values were 4.66 W, 93.12%, and 22.43, respectively. Maximum thermal stress is observed in square fin compared to the cylindrical fin.
3. Square fin demonstrates the highest efficiency among all. The efficiencies of the square, cylindrical, rectangular, trapezoidal, and triangular fins are found to be 94%, 93%, 91%, 92%, and 88%, respectively.
4. Fin performance depends on the perimeter. The rectangular fin achieves an effectiveness of 25.89 and a heat transfer rate of 5.42 watts, while the cylindrical fins show the lowest performance, with an effectiveness of 22.43 and a heat transfer rate of 4.68 watts.
5. For mild steel, stainless steel, cast iron, and titanium, the thermal resistances calculated were found to be 10.61, 32.55, 18.21, and 42.83 °C/W, respectively, corresponding to thermal conductivities of 46, 15, 26.8, and 11.4 W/mºC for a square cross section fin.

The results of the study go beyond mere trend description by revealing the underlying heat transfer mechanisms governing fin performance. The variation in temperature along the fin length, coupled with differences in heat transfer rate and efficiency across fin geometries, reflects the combined effects of conduction along the fin, convection to the surrounding air, and localized turbulence development, particularly around sharp edges and corners. The observed trends also carry scaling significance, as they provide insights that can be applied to larger or differently sized heat sinks, enabling designers to predict performance without extensive full-scale testing. From an engineering perspective, understanding how fin shape, material, and airflow behavior interact allows for the selection of optimal configurations that maximize heat dissipation while minimizing material usage and energy losses. These findings have direct practical implications for electronics cooling, compact heat exchangers, and industrial thermal management systems, guiding designers in creating efficient, cost-effective, and reliable thermal solutions.

Overall, experimental analysis validates simulations and theoretical models, ensuring real-world relevance, while simulations provide cost-effective testing. The integration of simulation and theory optimizes fin designs for better heat transfer efficiency. Theoretical analysis guides initial design decisions, and by comparing various fin designs and materials, the study identifies efficient solutions for heat dissipation and durability. Together, these methods offer engineers a comprehensive understanding for optimizing heat transfer applications.

### Scope for future work

1. Explore the performance of short fins under forced convection conditions and varying airflow velocities.
2. Investigate the impact of different heat flux levels and transient thermal loads on fin efficiency and effectiveness.
3. Examine the combined effect of fin geometry and material composites to enhance heat transfer and reduce weight.
4. Develop optimization algorithms integrating with the experimental data for automated fin design.

### Limitations of the study

1. The study considered a constant heat source, which may not fully represent variable or transient thermal loads in real applications.
2. Only short fins with specific cross-sectional geometries were analyzed; results may differ for longer fins or other shapes.
3. Material selection was limited to mild steel, stainless steel, cast iron, and titanium, excluding other advanced alloys or composites.
4. Long-term effects such as thermal fatigue, oxidation, or corrosion were not considered.

## Data Availability

The data that support the findings of this study are available from the corresponding author, Shiva Kumar, upon reasonable request.

## Abbreviations

AAV: Ambient air vaporizers
STIS: Shell and tube ice storage
PCM: Phase changing material
RSS: Root sum of squares
TTLHSS: Turbo tube heat storage system
FEA: Finite element Analysis
FOS: Factor of safety

## Variables

T: Temperature at any point on the fin at a distance x, ºC
T_a_: Ambient air temperature, ºC
T_o_: Root temperature, ºC
$\frac{dT}{dx}$: Temperature gradient, ºC/m
$x$: Distance along the fin, m
m: Fin factor, m^-1^
l: Length of the fin, m
h: Heat transfer coefficient, W/m^2^ ºC
k: Thermal conductivity, W/mºC
${\dot{Q}}_{fin}$: Heat transfer rate of fin, W
P: Perimeter of fin, m
A_c_: Area of cross section of fin, m^2^
A_s_: Surface Area, m^2^
*θ*: Temperature difference between temperature at any point of the fin and the ambient temperature, ºC
*θ*_o_: Temperature difference between root and ambient values,ºC
C_1_ and C_2_: *C*_1_ and *C*_2_ are arbitrary constants which are determined from the boundary conditions at the base and at the tip of the fin
$${\eta}_{fin}$$: Fin efficiency, %
*ε*: Fin effectiveness
U: Uncertainty analysis Function
W_R_: Total Uncertainty
x: Independent variable while determining the uncertainty
y: Uncertainty intervals
$\frac{\partial\left({\dot{Q}}_{fin}\right)}{{\dot{Q}}_{fin}}$: Total uncertainty present while evaluating heat transfer rate, %
$\frac{\partial\left({\eta}_{fin}\right)}{{\eta}_{fin}}$: Total uncertainty present while evaluating fin efficiency, %
$\frac{{\partial \left( \upvarepsilon \right)}}{\upvarepsilon }$: Error present while evaluating the fin effectiveness, %
h_1_/h_2_: Upper and lower bases
$\left[F\right]$: Force Vector matrix, W
$\left[K\right]$: Overall conductivity matrix, W/ºC
$\left[T\right]$: Temperature matrix,ºC
$\left[{K}_{c}\right]$: Conduction matrix, W/ºC
$\left[{K}_{h}\right]$: Convection matrix, W/ºC
$\left[{F}_{\stackrel{-}{Q}}\right]$: Heat generation vector matrix, W
$\left[{F}_{q}\right]$: Heat flux vector matrix, W
$\left[{F}_{h}\right]$: Boundary convection vector, W
$\stackrel{-}{Q}$: Internal heat generation, W/m^3^
$$q$$: Heat flux, W/m^2^

## Subscripts

a: Ambient
o: Root
c: Cross section
n: Intervals
h: Convection
1 & 2: Upper and lower
q: Flux
$\overline{Q }$: Internal Heat generation

## Greek letters

η: Fin Efficiency, %
ε: Fin Effectiveness
β: Coefficient of thermal expansion, 1/ºC
ν: Kinematic viscosity, m^2^/s
$$\delta$$: Change
